# “What will my child think of me if he hears I gave him HIV?”: a sequential, explanatory, mixed-methods approach on the predictors and experience of caregivers on disclosure of HIV status to infected children in Gombe, Northeast Nigeria

**DOI:** 10.1186/s12889-020-08506-x

**Published:** 2020-03-20

**Authors:** Oghenebrume Wariri, Ayomikun Ajani, Mercy Poksireni Raymond, Asabe Iliya, Olatoke Lukman, Emmanuel Okpo, Elon Isaac

**Affiliations:** 1 Vaccines and Immunity Theme, Medical Research Council (MRC) Unit, The Gambia at The London School of Hygiene and Tropical Medicine, Fajara, The Gambia; 2 Aberdeen Centre for Health Data Science (ACHDS), Institute of Applied Health Sciences, University of Aberdeen, Aberdeen, Scotland UK; 3 Department of Paediatrics, Federal Teaching Hospital (FTH), Gombe, Nigeria; 4 Department of Public Health Medicine, NHS Grampian, Aberdeen, UK; 5 Department of Paediatrics, College of Medical Sciences, Gombe State University, Gombe, Nigeria

**Keywords:** HIV, Disclosure, Barriers, Sequential, Explanatory mix-methods

## Abstract

**Background:**

With increasing access to effective Anti-Retroviral Therapy (ART), the proportion of children who survive into later childhood with HIV has increased. Consequently, caregivers are constantly being confronted with the dilemma of ‘*if*’, ‘*when*’, and ‘*how*’ to tell their children living with HIV their status. We aimed to determine the prevalence and predictors of disclosure and explore the barriers caregivers face in disclosing HIV status to children living with HIV in Gombe, northeast Nigeria.

**Methods:**

We conducted a sequential, explanatory, mixed-methods study at the specialist Paediatric HIV clinic of the Federal Teaching Hospital Gombe, northeast Nigeria. The quantitative component was a cross sectional, questionnaire-based study that consecutively recruited 120 eligible primary caregivers of children (6–17 years) living with HIV. The qualitative component adopted an in-depth one-on-one interview approach with 17 primary caregivers. Primary caregivers were purposively selected to include views of those who had made disclosure and those who have not done so to gain an enhanced understanding of the quantitative findings. We examined the predictors of HIV status disclosure to infected children using binary logistic regression. The qualitative data was analysed using a combined deductive and inductive thematic analysis approach.

**Results:**

The mean age of the index child living with HIV was 12.2 ± 3.2 years. The prevalence of disclosure to children living with HIV was 35.8%. Children living with HIV were 10 times more likely to have been told their status if their caregivers believed that disclosure had benefits [AOR = 9.9 (95% CI = 3.2–15.1)], while HIV-negative compared to HIV-positive caregivers were twice more likely to make disclosures [AOR = 1.8 (95%CI = 0.7–4.9)]. Girls were 1.45 times more likely than boys to have been disclosed their HIV positive status even after adjusting for other variables [AOR = 1.45 (95% CI = 0.6–3.5)].

Caregivers expressed deep-seated feeling of guilt and self-blame, HIV-related stigma, cultural sensitivity around HIV, and fears that the child might not cope as barriers to non-disclosure. These feeling were more prominent among HIV-positive caregivers.

**Conclusion:**

The process of disclosure is a complex one and caregivers of HIV positive children should be supported emotionally and psychologically to facilitate disclosure of HIV status to their children. This study further emphasises the need to address HIV-related stigma in resource constrained settings.

## Background

The number of new HIV infections among children has decreased globally due to effective preventive strategies such as the prevention of mother to child transmission (PMTCT) of HIV [1]. Nevertheless, Nigeria still has the second largest HIV epidemic globally and contributes almost a third of paediatric HIV infections worldwide [2]. By 2017, with 380,000 children living with HIV infection, Nigeria had the largest burden globally, with a quarter of this number being on ART [3].

With increasing access to effective ART which has reduced HIV-related deaths by 42% since the year 2000, the proportion of children who survive into later childhood and live chronically with HIV has increased [4]. Consequently, caregivers of children living with HIV are constantly being confronted with the dilemma of ‘*if*’, ‘*when*’, and ‘*how*’ to tell these infected children of their HIV status [5, 6]. Disclosure of HIV to infected children poses serious implications for the child and their caregivers that relate to treatment adherence, adjusting to the illness at different stages of life such as entering adolescence and relationship with the wider community [7].

Disclosure of HIV to infected children is a critical component of the process of living with HIV and is considered pivotal to the continuum of care [6, 8]. Disclosure is considered to have been done when a child has been fully informed of his or her HIV status and if the terms HIV, AIDS, or any local term specifically associated with HIV/AIDS had been used in a discussion with the child about their health [6]. Rates of disclosure in children in sub-Saharan Africa vary widely, ranging from 0 to 69.2%, [9] despite the World Health Organization (WHO) recommending in 2011 that children from 6 to 12 years living with HIV should be gradually informed about their status in an ongoing, age-appropriate manner [6]. This may be related, but not limited to a complex social and cultural barrier regarding how child-caregiver relationships are conceptualised in the context of healthcare, with children expected to be compliant recipients of services, while the caregiver interacts with the health system on their behalf [5].

There is evidence to suggest that it is beneficial to disclose HIV status to infected children before they reach adolescent as it fosters ART adherence, participation in ongoing care, and psychological resilience, while lessening the risk of horizontal transmission due to risky sexual behaviour [9, 10]. Most research on disclosure of HIV to infected children has mainly been quantitative focusing on the determinants of disclosure, patterns of disclosure, the differences between non-disclosers and disclosers, and the impact of disclosure on certain HIV outcomes in children and adolescents [9]. It is, however, difficult to have an enhanced understanding of the ‘*why’*, and the ‘*how*’ behind the evidence in terms of why disclosure is done or not done, and how disclosure is conceptualized by caregivers and their children living with HIV [6, 9]. Gaining an enhanced understanding of the contextual and granular issues behind the ‘*numbers*’ would be pivotal in planning, implementing and evaluating strategies to scale up childhood disclosure and aiding improved childhood age-appropriate engagement in HIV/AIDS care.

Mixed method approach; a combination of quantitative and qualitative research paradigms produces an enhanced understanding and granular evidence in comparison to ‘*mono-methods*’ research [11, 12]. There are limited African studies that have used mixed methodologies to explore the complex and context-specific issue of disclosing HIV to children living with HIV [5, 13, 14]. Combining research methods draws on the commonalities of both paradigms and harnesses their differences in a complementary manner to generate robust research evidence, which may contribute to the understanding of a complex phenomenon like disclosure [12]. This research, therefore, was designed with the foregoing in mind, drawing on the strengths of both paradigms to gain enhanced understanding of HIV disclosure to children living with HIV. Our aim was to determine the prevalence and predictors of disclosure and explore the barriers caregivers face in disclosing HIV status to infected children in Gombe, northeast Nigeria.

## Methods

### Study setting and population

The study was conducted at the Federal Teaching Hospital, Gombe, a 450-bed capacity tertiary health facility located in Gombe State, northeast Nigeria. Gombe State is strategically located at the centre of the northeast sub-region of Nigeria between latitudes 90°30’ and 12°30’N and longitudes 8°5’ and 11°45’ E and has a population of 2.4 million people, with children 18 years and below being approximately 50% of the population and has a projected population growth rate of 2.3% per year [15]. The HIV sero-prevalence of 3.4% in Gombe State is identical to the National sero-prevalence rate [16].

Established in the year 2000, the Federal Teaching Hospital Gombe serves as the major referral centre for hospitals within the state and from neighbouring states of the northeast region of Nigeria, which has a combined population of 23,558,674 [15]. The facility has a specialised paediatric HIV/AIDS clinic operational since the inception of the hospital and serves about 200 children from birth till they turn 18 years. Services offered include PMTCT, HIV counselling and testing (HCT), monitoring/follow-up of patients including viral load; treatment of opportunistic and other infections and provision of ART. Clinics are held once a week and about 30 children usually accompanied by their primary caregivers are consulted during clinic days. The clinic is run by a consultant paediatrician (EI) specialised in HIV/AIDS care, supported by four paediatric trainees, five specialist nurses, laboratory and pharmacy staff on clinic days.

### Study design

**Fig. 1:**
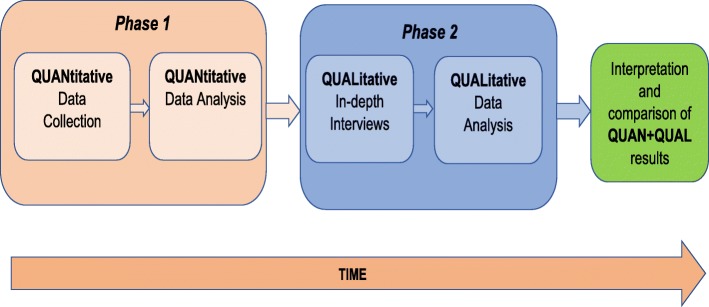
A visual representation of the sequential, explanatory, mixed-methods design adopted in this study

This was a sequential, explanatory, mixed-methods study (Fig. 1) that used a combined quantitative and qualitative research design in two distinct phases. The initial phase (phase 1) was a quantitative design, was cross-sectional descriptive in nature and was questionnaire based. In the next phase (phase 2) which was conducted to further explain and enhance our quantitative findings, we adopted a qualitative design that involved in-depth interviews of primary caregivers of HIV-positive children.

### Participants

All primary caregiver responsible for the care of children aged 6–17 years living with HIV and attending the specialist paediatric HIV clinic of the Federal Teaching Hospital Gombe, Nigeria, who consented to the study were eligible for inclusion in the study. We defined a *primary caregiver* as an adult aged ≥18 years attending our specialist clinic with their HIV-positive child, and responsible for the day-to-day care of the child (6–17 years), including but not limited to biological parents.

We chose caregivers of children of aged 6–17 years [those with the cognitive skills and emotional maturity of a normally developing child of 6–17 years] because the WHO recommends that children of this age group should have disclosure of their HIV status made to them in an age-appropriate manner [6]. HIV positive children who did not have an identified primary caregiver were excluded from the study. One hundred and twenty-five (125) out of the 200 primary caregivers attending the specialist clinic with their HIV-positive children gave their consent and were recruited into the study. Participants were advised that they could withdraw from the study at any point.

### Data collection

All data for this study was collected between October 2017 and September 2018.

#### Phase 1: quantitative component

A pre-tested questionnaire (Additional file 1), developed specifically for the purposes of this study was administered to primary caregivers of HIV-positive children in the absence of the child. The questionnaire was developed based on our understanding of the study population and after reviewing the literature on predictors of disclosure of HIV status to infected children in the setting of sub-Saharan Africa. The questionnaire captured information on caregivers’ sociodemographic characteristics, their relationship with the index child, disclosure status of the index child, opinion about the process of HIV disclosure and information about the care and management of the HIV-positive child. Disclosure was considered to have been done when a child had been fully informed of his or her status and if the terms HIV, AIDS, or any local term specifically associated with HIV/AIDS had been used in a discussion with the child about their health [6]. The quantitative data was analysed after phase 1 and the findings informed the next sequential phase which was conducted to give an in-depth understanding and explanations for disclosure and non-disclosure among primary caregivers (Fig. 1).

#### Phase 2: qualitative component

In-depth one-on-one qualitative interviews were conducted with 17 primary caregivers who were selected purposively from the 125 primary caregivers who met the eligibility criteria. Caregivers who were interviewed were selected specifically based on whether or not they had disclosed to the child his/her HIV status. (Table 1). We aimed to maximize diversity with respect to caregivers’ HIV status, thus, we included HIV positive and HIV negative caregivers to provide multiple perspectives and lived experiences. Using this approach, we sought to gain an in-depth understanding through the lived experiences of caregivers, the reasons why most caregivers in our setting were reluctant to make disclosure to their children living with HIV.

**Table 1:** Characteristics of the 17 caregivers interviewed in the qualitative component of the study

Caregiver code no	Type of caregiver	Caregiver HIV status	Child’s age category*	Orphan?	Disclosure done?
01	Uncle	Negative	Mid adolescence	Yes	**Yes**
02	Mother	Positive	Mid adolescence	No	**Yes**
03	Mother	Positive	Early adolescence	No	**No**
04	Adopted mother	Negative	Early adolescence	Yes	**No**
05	Mother	Positive	Early adolescence	Yes	**Yes**
06	Mother	Positive	Early adolescence	No	**No**
07	Mother	Positive	School age	No	**No**
08	Aunt	Negative	Early adolescence	Yes	**Yes**
09	Mother	Positive	School age	No	**No**
10	Aunt	Negative	Early adolescence	Yes	**Yes**
11	Mother	Positive	School age	No	**No**
12	Father	Positive	Mid adolescence	No	**Yes**
13	Father	Positive	Late adolescence	No	**Yes**
14	Mother	Positive	School age	No	**No**
15	Father	Positive	Mid adolescence	No	**Yes**
16	Father	Positive	Early adolescence	No	**No**
17	Father	Positive	Early adolescence	No	**No**

**Note:** Caregivers’ gender, parent vs non parent caregiver, HIV positive/ negative status, varying levels of education and religious backgrounds were other characteristics that were considered in purposively selecting caregivers for the in-depth interviews in addition to the main characteristics of whether or not caregivers have made disclosure to their children. *School age (6–9 years), Early adolescence (10–13 years), Mid adolescence (14–16 years) and Late adolescence (17–18 years) [17].

Interviews were held at times and locations determined by participants to be convenient for them. Interviews were semi-structured, used topic guides (Additional file 2) and were conducted by co-authors (AA, MPR and AI) who had previous experience of conducting qualitative interviews and facilitating focus group discussions. One interview each was conducted per primary caregiver and lasted about 60–90 min. Interviews were conducted in either *Hausa* (the predominant language spoken in northern Nigeria) or English language based on which language the participants preferred. Interviews conducted in *Hausa*, were professionally transcribed verbatim into Hausa language, before being translated to English. Narrative data were then cross-checked for trustworthiness by a co-author (co-author AA) who could read, write, and communicate fluently in *Hausa* by listening to the audio recording of interviews in *Hausa* repeatedly while reading through transcribed text in English.

### Data analysis

All quantitative data generated were processed and analysed using the IBM Corp SPSS statistics for windows version 24.0 (Armonk, NY: IBM Corp). Proportions, and percentages were reported for categorical variables, while mean and standard deviations were reported for continuous variables which were normally distributed.

To determine which caregiver or child’s characteristic (the independent variables) that was predictive of disclosure of HIV status to infected children (the dependent variable), multiple logistic regression models were fitted for variables that were significantly associated with disclosure from the univariate analysis. The regression models were adjusted for confounders including age of the index child, duration the index child has been diagnosed HIV-positive and how long the index child has been on ART. The adjusted odds ratio (AOR), and 95% CI are reported, with significance level set at *p*-values of < 0.05.

Qualitative narrative data generated from transcripts of in-depth interviews were imported into the QSR NVivo software version 10 (QSR International Pty Ltd. Version 10, 2012) and analysed thematically using the approach described by Braun and Clark [18]. The thematic analysis was based on a combined inductive and deductive approach [12]. The inductive approach determined meanings that emerged from within the data, while the deductive approach looked for categories and meaning within the data that were determined a priori based on evidence from literature*.* Researchers (OW and AA) separately read the transcripts several times to familiarize themselves with the key ideas, paying attention to recurring themes or patterns. Initial themes and their sub–themes were noted as codes. Transcripts were again re–read, re–checking for themes, how new themes supported the data and vice versa, identifying relationships within and between themes. This process was followed in an iterative manner until thematic saturation was reached. The emergent themes and sub-themes were then discussed and agreed on collectively by co-authors, and where there was a disagreement on a theme or sub-theme, a third person (a co-author) was consulted to help resolve the disagreement.

## Results

### Quantitative findings

#### Primary caregivers characteristics and opinion on disclosure to HIV-infected children

A total of 125 caregivers were recruited to this study. Of these, data was incomplete or missing for 5 participants leaving 120 participants that were included in the final analysis. There were 98 (81.7%) female caregivers, giving a female to male caregiver ratio of 4.5:1. The mean age of primary caregivers included in the study was 39.2 ± 9 years. Majority [77.5% (93/120)] of caregivers were the biological parents of the HIV positive child. Forty seven percent of caregivers were educated up to the tertiary level. Majority, 77.5% (93/120) of caregivers believed that disclosure had benefits and should be made to children living with HIV (Table 2).

**Table 2:** Characteristics of 120 caregivers of children living HIV with included in quantitative component of the study

Variable	Characteristics	Frequency (n)	Percentage (%)
Gender	Male	98	81.7
	Female	22	18.3
Educational level	None	18	15
	Primary	15	12.5
	Secondary	30	25
	Tertiary	57	47.5
Relationship with Child	Biological parent	93	77.5
	*¶*Other family member	27	22.5
Caregiver’s HIV status	Positive	94	78.3
	Negative	26	21.7
Opinion about telling a child his/her HIV status	Agree to disclose	93	77.5
	Disagree to disclose	27	22.5
Opinion on benefits of telling a child his/her HIV status	No benefits	17	14.2
	There are benefits	103	85.8
Opinion about appropriate age of disclosure	School age	5	4.2
	Early adolescence	31	25.8
	Mid adolescence	53	44.2
	Late Adolescence	31	25.8

Values presented as mean (SD), unless otherwise indicated, **n (%) =** Frequency (percentage)

School age (6–9 years), Early adolescence (10–13 years), Middle adolescence (14–16 years), Late adolescence (17–18 years) as described by Cromber et al. [17]

¶Others: Sister, Brother, Neighbour, Step-mother, Guardian, and unknown person

#### Characteristics of index children living with HIV

The mean age of index children living with HIV was 12.2 ± 3.2 years and there was no statistically significant difference between the ages of boys and girls with *p* = 0.363 (Table 3). There were 60 (50%) Boys, making a Boys to Girls ratio of 1:1. The mean age at first diagnosis of HIV in these children was 4.9 ± 3.7 years, while the mean duration of treatment with ART was 7.0 ± 3.9 years, with no significant differences in boys and girls (Table 3). Most, 98.3% (118/120) of children were on ART.

**Table 3:** Characteristics of index children living with HIV (*N* = 120) included in the quantitative component of the study

Variable	Boys (***N*** = 60)	Girls (***N*** = 60)	***p*** value	Total (***N*** = 120)
***Age (years)***	11.9 (2.9)	12.5 (3.5)	0.363*	12.2 (3.2)
***Age at 1st diagnosis (years)***	5.1 (3.8)	4.6 (3.5)	0.435*	4.9 (3.7)
***Duration of ART treatment (years)***	6.6 (3.4)	7.6 (4.2)	0.98*	7.0 (3.9)
***Overall Age group: n (%)***				
School age	11 (18.8)	11 (18.3)		22 (18.3)
Early adolescence	25 (41.7)	33 (55.0)		58 (48.3)
Mid adolescence	15 (25.0)	11 (18.3)		26 (21.7)
Late adolescence	9 (15.0)	5 (8.3)	0.472**	14 (11.7)
***School Attendance: n (%)***	60 (100)	58 (96.7)	0.476**	118 (98.3)
***On Antiretrovirals: n (%)***	59 (98.3)	58 (96.7)	0.559**	117 (97.5)
***Prevalence of disclosure: n (%)***	18 (30.0)	25 (41.7)	0.253**	43 (35.8)
***∫Disclosure rate by age-group: n (%)***				
School age	1 (9.1)	2 (18.2)		3 (13.6)
Early adolescence	3 (12.0)	10 (30.3)		13 (22.4)
Mid adolescence	6 (40.0)	8 (72.7)		14 (53.8)
Late adolescence	8 (88.9)	5 (100)	0.001**	13 (92.9)
***Who made disclosure: N = 43 n (%)***				
Mother alone	7 (38.9)	8 (32.0)		15 (34.9)
Father alone	0 (0.0)	5 (20.0)		5 (11.6)
Health worker	4 (22.2)	9 (36.0)		13 (30.2)
Grandmother	2 (11.1)	1 (4.0)		3 (7.0)
*¶*Others	5 (27.8)	2 (8.0)	0.212**	7 (16.3)

Values presented as mean (SD), unless otherwise indicated, **n (%) =** Frequency (percentage), *Independent t-test p-values reported, **Chi-square p-values reported

**∫**Disclosure rate by age-group calculated based on overall number of children within each age group category (disclosed and non-disclosed)

¶Others: mother and father combined, Sister, Brother, Neighbour, Step-mother, Guardian, and unknown person

School age (6–9 years), Early adolescence (10–13 years), Middle adolescence (14–16 years), Late adolescence (17–18 years) as described by Cromber et al. [17]

#### Prevalence of disclosure of HIV status to infected children

The prevalence of HIV status disclosure to infected children was 35.8% (43/120). More mothers, 34.9% (15/43) compared to fathers, 11.6% (5/43) had made disclosures, however, the differences were not statistically significant (*p* = 0.212). Disclosure rate was higher in girls compared to boys, 41.7% vs 30%, *p* = 0.253 (Table 3).

#### Predictors of disclosure of HIV status to infected children

**Fig. 2:**
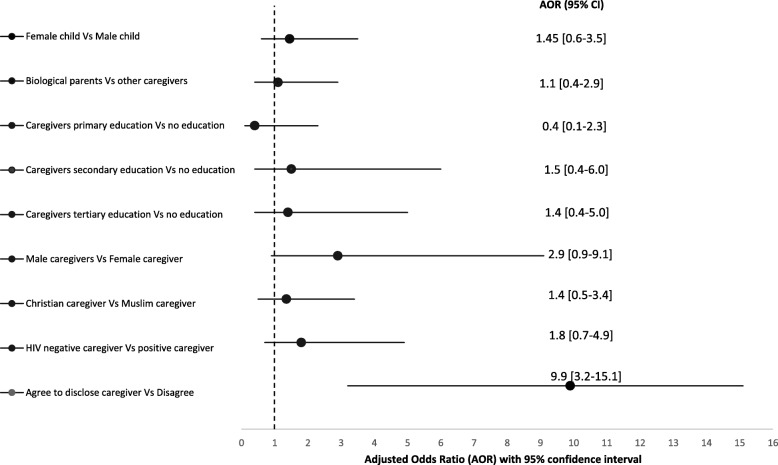
Comparative odds ratios for likelihood of telling a child his/her HIV status by caregiver and child’s characteristics. Note: all variables were adjusted for child’s age, duration (in years) the child has been on HAART, and duration (in years) since the child was first diagnosed with HIV

HIV-positive children were ten times more likely to have been told their status if their caregivers believed that disclosure was beneficial and should be made to children living with HIV [AOR = 9.9 (95% CI = 3.2–15.1)]. Children’s gender, caregiver’s religion, and whether the caregiver was the child’s biological parent or not did not predict disclosure (Fig. 2). Male caregivers compared to females were 3 times more likely, while HIV-negative caregivers compared to HIV-positive caregiver were 2 times more likely to have made disclosure to their children, however, the odds were not statistically significant (Fig. 2). Girls were 1.45 times more likely than boys to have been disclosed their HIV positive status even after adjusting for other variables [AOR = 1.45 (95% CI = 0.6–3.5)].

### Qualitative findings

Three main themes; a) barriers to disclosure; b) the process of disclosure; and c) the perceived impact of disclosure on the child arose from the qualitative data related to why most caregivers have not made disclosure to their children living with HIV. The emergent sub-themes within the three themes (Table 4) are presented below and supported with verbatim quotes.

**Table 4:** Major emergent themes and sub-themes from the qualitative data

Themes	Sub-Themes
**Barriers**	Stigma and discrimination
	Blame, guilt and responsibility
	Fear of the unknown
	The ‘right time’
	Multi-layer disclosure of secrets
	Cultural sensitivity
**Process**	Ongoing and gradual
	Other peoples’ or shared responsibilities
**Impact**	Mutually empowering
	Intimacy and peer support

### Barriers

#### Stigma and discrimination

There was consensus among parents, in a recurring manner across the interviews that discrimination and stigma were major deterrents to disclosure of HIV to their infected children. They opined that stigmatization in the community was still quite high and expressed deep-seated fears of what appeared to be the community’s way of labelling and ostracizing HIV positive individuals. The discussion highlighted their fears of the negative impact of stigma and discrimination on their wards and they were reluctant to expose their children to such; fearing the impact it could have on them, hence they would rather resort to non-disclosure.

“…honestly when he tells someone, almost the entire area [community] will come to know he is positive and knowing his status, they will start discriminating against him and when he goes somewhere, he may not be welcomed…at the time they start discriminating him he will feel hated and will prefer to die” (**Caregiver 1**: HIV negative caregiver of HIV orphan)

“…when other children hear about it [the child’s HIV status], they will start to disassociate themselves from him. These are my reasons why a child should not be disclosed his HIV status” (**Caregiver 6**: HIV positive caregiver of adolescence living with HIV)

#### Blame, guilt, and responsibility

The caregivers shared the view that while already having to deal with the pain of watching their children grow-up HIV positive, disclosure was made more complex by thoughts of questions that would arise during the process. The caregivers in a repetitive manner harboured strong feelings of guilt and self–recrimination; anticipated being blamed and despised by their children for what they unwittingly brought upon them, and some considered the possibility of loss of the respect they have from their children, hence preferring non-disclosure. These views were more prominently expressed by HIV positive caregivers.

"This [disclosure] can lead him to query his parents from where and how they came about the disease…He may think his parents are irresponsible and it led to them contracting HIV, so you see the parent will not want to tell him because they fear they may lose the respect he has for them. So, this makes disclosure problematic for the parents. (**Caregiver 2**: HIV positive caregiver of adolescent living with HIV)

“I don’t know what my child will think of me if he hears I gave him HIV. He may say I have harmed him because it is like harming him by infecting him with the virus.” (**Caregiver 7**: HIV positive caregiver of a school age child living with HIV)

The main reason parents will keep it a secret, like I told you, is the fear of what the children will think of them and how they look at them once they discover that their parents have the disease [HIV]…they will begin to ask themselves how their father became infected… is it through sex? (**Caregiver 17:** HIV positive caregiver of an adolescent living with HIV)*.*

#### Fear of the unknown

Many caregivers feared that their children could not handle the news and their situation could be worsened following disclosure of their HIV positive status. Their uncertainty about the impact of this news on children who had grown up seemingly unaware of any problems could be felt across the discussions repeatedly. They expressed fears about this knowledge resulting in depression, anxiety, hopelessness and even suicidal thoughts in their wards.

“…he may resolve to take something [poison] to end his life because he will believe when told that he is HIV positive that his life is of no importance or that his parents have cheated him by bringing him into the world with this illness” (**Caregiver 5**: HIV positive caregiver of adolescent living with HIV)

“I keep wondering what her state of mind will be when I finally confide in her” (**Caregiver 14**: HIV positive caregiver of a school age child living with HIV)

#### The ‘right time’

During discussions, it became apparent that 14–15 years and above was deemed to be the ‘right time’ to begin disclosure, thus, having younger children acted as a barrier to disclosure. Most caregivers perceived that at that age, the child would have attained the ability to process such information. Other caregivers’ opinion was that since girls began to mature earlier than boys, the process should begin earlier in them.

“The child doesn’t have a right to know his or her status till age 15years. It is when they reach 15 years that the time is right for them to know their status and condition, they are in…” (**Caregiver 11:** HIV positive caregiver of a school age child living with HIV)

“…in primary school between 7,8,9 to 10 years it may be difficult- for instance for female you can tell her from 10 years at least she has grown up…However, a male child can wait until when he is like 14, 15 years…” (**Caregiver 1**: HIV negative caregiver of an HIV orphan)

#### Multi-layer disclosure of secrets

In a repeated manner, the caregivers emphasized the secrecy surrounding being HIV positive or having an HIV positive family member in their local context. Anxiety about knowledge of the child’s and possibly parent status subsequently becoming known to the larger community through the child following disclosure was palpable. This concern appeared to be a primary contributor to most parents suggesting an optimal disclosure age purely based on when the child would be able to keep his/her “mouth shut”.

“…they will begin to say it outside and the neighbours may ask you in order to clarify the information, and it is from there that others will get to know the family’s secret” (**Caregiver 7:** HIV positive caregiver of a school age child living with HIV)

‘The children should be told that they can only tell a select few people who are also positive and who can keep this information secret, so that they will know that it is not everywhere you go talking about your status". (**Caregiver 16**: HIV positive caregiver of a school age child living with HIV)

#### Cultural sensitivity

There was a recurring theme during the discussions on the impact of cultural practices or beliefs on the disclosure process especially on issues relating to HIV and sexuality. Caregivers agreed there was a tendency to shy away from discussing topics relating to sex with their wards and admitted this presented a huge barrier to disclosure.

“Assuming it is in our culture to always give answers to whatever [including sexual topics] a child asks by telling them the truth; I don’t think there will be problems in disclosing HIV to our children” (**Caregiver 2**: HIV positive caregiver of adolescent living with HIV)

### Process of disclosure

#### Ongoing and gradual

The caregivers mostly agreed that disclosure needed to be done gradually, as a stepwise and ongoing process. They agreed that a process of gradually breaking the news over a long period would help the child cope better.

“For a difficult issue like HIV, disclosure has to be gradual, systematic and continuous…it has to be slow, phase by phase” (**Caregiver 17:** HIV positive caregiver of an adolescent living with HIV)*.*

#### Other peoples’ or shared responsibilities

Most of the caregivers considered the process of disclosure as ‘other peoples’ or a ‘shared’ responsibility, believing that health care workers should play a key role in the process; either as facilitators or to provide support while caregivers make disclosure. Some, especially the HIV positive female caregivers emphasized parental responsibility to make disclosure or pointed fingers at fathers as responsible, blaming them for infecting the rest of the family.

“The responsibility lies on both the parents and the doctors. The parents are the ones that know the problem of their children…On the other hand, the doctors can disclose to him because they are the ones that see him and prescribe medicine for him, they tell him the steps to take to protect his health…” (**Caregiver 11:** HIV positive caregiver of a school age child old living with HIV)

“The father has the responsibility of disclosing the child’s HIV status to him since he is the person that brought the disease home/ into the family” (**Caregiver 5**: HIV positive caregiver of an adolescent living with HIV)

### Impact of disclosure

#### Mutually empowering

Full disclosure was repeatedly described as associated with feelings of relief for the caregivers and liberating for the child throughout the discussions. Involvement of children in HIV management following disclosure was a major positive effect observed by caregivers who had made disclosure.

“I saw clearly that if I left him to grow much older before telling him, he is likely to say I have been unfair to him by keeping him in darkness/ hiding things from him. We both have peace of mind since he came to understand the illness” (**Caregiver 8:** HIV negative caregiver of an adolescent HIV orphan)

“When the doctor disclosed it to him, I saw some changes in his behaviour and noticed he was more serious about committing himself to taking his drugs more than before” (**Caregiver 13**: HIV positive caregiver of an adolescent living with HIV)

#### Intimacy and peer support

Caregivers described how full disclosure positively impacted on their child or ward as it brought intimacy and an opportunity to get support from family members and peers in school who were dealing with similar situations or had someone who was also HIV positive. This intimacy or support was not possible prior, as the child’s condition was kept secret.

“He later told me that in school he has a friend whom he confided in about his HIV status and who also told him about his sister at home who had HIV. You see that person also knows about the disease from personal experience, so he won’t go about telling people that my son is infected …and he can now support my son” (**Caregiver 2**: HIV positive caregiver of an adolescent living with HIV)

## Discussion

The strength of our study was that it combined quantitative and qualitative methodologies in a sequential and complementing manner to gain in-depth information on the predictors, and experience of caregivers on the disclosure of HIV status to infected children attending an HIV-specialist clinic in the northeast of Nigeria. Thirty-six percent of the HIV positive children in this study have had their HIV status formally disclosed to them by their caregivers. This figure is much higher than an earlier report by Brown et al. (13.5%) in Ibadan, South-West Nigeria, and may be attributed to differences in study population, context, and increasing awareness about HIV disclosure [19]. For example, most children studied in the earlier report were pre-adolescent (mean age 8.8 ± 2.2 years) compared to our study in which the majority were in early and mid-adolescence (mean age 12.2 ± 3.2 years), making disclosure more likely in our study with corresponding higher prevalence. More recent studies in Nigeria by Ubesie et al., [20] and Odiachi et al. [21] reported prevalence of disclosure to HIV positive children of 29.5 and 30.9% respectively; comparable to our findings which suggests an increase prevalence of disclosure to Nigerian children living with HIV within recent time. Although relatively higher than most reports from within Nigeria and the African continent, [5, 22–28] our disclosure rate are lower compared with findings from the US, Canada and Europe [29–31].

Majority of caregivers appeared to support the idea of disclosing HIV to positive children as necessary however, up to 70% of them considered that it should be left till mid adolescence when the child would have become “mature”. There was concordance between the quantitative and qualitative components of our study with both showing that caregivers preferred 14–15 years as the average age to tell children they were HIV infected. In a similar pattern, John-Stewart et al. [32] reported that although 79% of Kenyan caregivers answered yes to the question “is it important for a parent/caregiver to inform a child of his/her status?” only 19% had disclosed, and the preferred age of disclosure was 12 years. The preferred age for commencement of disclosure by the caregivers we studied is both high and is discordant with Nigerian National guidelines, [33] the recommendations by the African Network for the Care of Children Affected by HIV/AIDS (ANECCA), [34] and WHO [6] which encourage beginning the process of disclosure for school aged children. This discordance between the preferred age of disclosure in our study to national and international guidelines is disturbing. It could be due to the caregivers’ perception of when the child is psychologically and emotionally mature and has gained relevant cognitive skills to deal with the disclosure. It further highlights the perception that disclosure should be a ‘one-time’ event in adolescence rather than a stepwise and gradual process [35] and implies that children in our study setting are likely to learn of their HIV-status suddenly and at an older age. This could have critical implications for the continuation of HIV care.

In contrast to other reports, [20, 26] girls were 1.45 times more likely than boys to have been disclosed their HIV positive status. This quantitative finding was corroborated by the qualitative component of our study where caregivers shared their views that female children tend to attain ‘maturity’ earlier than their male counterparts and would therefore be better equipped to cope with the news, thus, explaining the higher likelihood of disclosure to female children in our study setting. Although biological and psychosocial models of development may support the assertion by the caregivers in our study that girls attain physical and psychological maturity earlier than boys, in the context of a complex condition like HIV, ensuring that caregivers are supported to make disclosure to their boys and girls alike is critical to effective treatment uptake.

Although not an independent predictor of disclosure, having secondary or tertiary education in our study was associated with a higher likelihood of disclosure to HIV positive children. Other reports have shown conflicting reports on the influence of caregivers’ educational status on disclosure to HIV infected children with some suggesting that attainment of higher levels of education was negatively associated with disclosure to children [36, 37]. This quantitative finding was further explained by the observation in the qualitative component of the study that local traditions, more prevalent among non-educated caregivers discourage communication on sexuality and HIV infection between parents and their children. There is therefore a need to promote and encourage open discussions across families in the Nigerian context more broadly irrespective of their educational background, and specifically among families affected by HIV.

In our study, caregivers who believed that disclosure was beneficial and should be made to their children living with HIV were ten times more likely to disclose the HIV status to the child. Similar findings have been reported by other studies that have found higher disclosure rates among children of caregivers that understood the potential health benefit of disclosure [23, 31, 38]. This, therefore, underscores the need for a systematic approach to providing training, guidance, and support for caregivers on disclosure to HIV positive children especially in resource constrained settings with the hope of encouraging them to believe in disclosure, thus, increasing disclosures to children across context.

Furthermore, our study found that HIV negative caregivers were twice more likely to disclose HIV status to their infected children or wards compared to caregivers who were HIV positive. This quantitative finding may be explained by evidence from the qualitative component of our study which indicated that deep seated feeling of shame, guilt, self-blame, and self-recrimination among HIV-positive caregivers strongly influenced disclosure practices due to their perceived role in unknowingly transmitting HIV to their children during pregnancy, childbirth and after childbirth. HIV negative caregivers might be less likely to face these feelings when confronted with making disclosures, with a correspondingly higher likelihood of making disclosure to their HIV infected children or wards. Similar to our findings, Kiwanuka et al. reported that Ugandan HIV positive caregivers reported fears of being considered to have been promiscuous or irresponsible by their children following the disclosure of HIV status to their infected children [39]. These findings suggest that the psychological burden of being responsible for the child’s illness partly impedes the disclosure process by caregivers. Consequent upon this quantitative finding whose understanding was enhanced by the qualitative data, health care workers providing care to HIV positive children and their caregivers must therefore understand these undertones to enable them to provide supportive and empathetic care.

The qualitative data in our study showed that stigma and discrimination were major caregivers’ concerns preventing disclosure to HIV positive children. Some parents felt their children might harm them or commit suicide when they became aware of their HIV status. The high level of stigma and discrimination against people living with HIV (PLHIV) in many communities across Nigeria which links HIV infection with promiscuity may perpetuate parental fears of being despised by their wards following disclosure [40, 41].. Fear of stigma also fuels the desire of parents to maintain the HIV status of family member’s secret - a factor that militates against early disclosure to children for fear of inadvertent disclosure. Activities aimed at reducing stigma and discrimination within communities should be strengthened in order to reduce this trend.

Disclosure was mostly described as mutually beneficial for caregivers and children studied. In consonance with many other reports, [19–21, 42, 43] this study showed that major benefits accrued from telling children the truth about their HIV status which included relief for caregivers and children, improved commitment to involvement in medication and the opportunity to develop peer support. These benefits further highlight the importance of disclosure thus, strategies aimed at encouraging caregivers to make disclosures and supporting them through the disclosure process should be scaled up by health systems with similarities to our study context.

Our study has some limitations. Firstly, the fact that we relied on caregivers’ reported information to determine if disclosure has been made could have increased the likelihood of reporting bias. This may have decreased or increased our reported prevalence of disclosure. However, the quantitative questionnaire was researcher administered and the fact that an operational definition [6] was used to define disclosure could have minimized the possibility of this bias. Secondly, we interviewed majorly women, and HIV positive caregivers which could have influenced the perspectives and experience shared in the qualitative narrative data. However, our purposive sample represent our study setting and therefore, appropriately reflect the experience from our setting. One should be cautious when extrapolating our qualitative findings to other settings with different context.

## Conclusion

This sequential, explanatory, mixed-methods study provides rich and granular research evidence that enhances the understanding of the barriers, predictors, experience and the dilemmas caregivers face in disclosing HIV status to infected children in a resource limited setting. HIV status disclosure to infected children was found to be low in our study setting. Disclosure was affected by caregivers’ guilt and self-blame, HIV stigma, cultural sensitivity around HIV infection, the child’s age/gender, caregivers’ belief on the importance of disclosure, and caregiver’s HIV status. Despite the pervasive barriers, disclosure was mostly described by caregivers as mutually beneficial for caregivers, children, and the continuum of HIV care. There is, therefore, a critical need to develop context-specific interventions to support caregivers who face multiple barriers in disclosing HIV status to infected children in our study setting. Such interventions could be delivered through the routine HIV treatment and care system by health workers who are trained and supported by locally-relevant guidelines based on local evidence. Through this process, caregivers would be empowered with practical skills needed to recognise windows of opportunities to initiate disclosure early and manage the process in a manner appropriate to their children’s physical and emotional development.

## Supplementary information

**Additional file 1.** Pretested quantitative data collection questionnaire

**Additional file 2.** Qualitative interview guide

## Data Availability

The datasets analysed during the current study are available from the corresponding author on reasonable request.

## Abbreviations

AIDS: Acquired Immune-deficiency Syndrome
ANECCA: African Network for the Care of Children Affected with HIV/AIDS
AOR: Adjusted Odds Ratio
ART: Anti-Retroviral Therapy
CI: Confidence interval
HAART: Highly Active Anti-Retroviral Therapy
HCT: HIV Counselling and Testing
HIV: Human Immune-deficiency Virus
PLHIV: People Living with HIV
PMTCT: Prevention of Mother To Child Transmission of HIV
SD: Standard deviation
WHO: Word Health Organization

## References

[1] UNAIDS (2015). How AIDS changed everything. MDG 6: 15 Years, lessons of hope from the AIDS Response.

[2] National Agency for the Control of AIDS (NACA) (2017). National Strategic framework on HIV and AIDS: 2017-2021.

[3] United Nations Children’s Fund (UNICEF) (2017). UNICEF Annual Report 2017 Nigeria.

[4] UNAIDS (2015). World AIDS Day. AIDS by the numbers.

[5] Vaz LM, Maman S, Eng E, Barbarin OA, Tshikandu T, Behets F (2011). Patterns of disclosure of HIV status to infected children in a sub-Saharan African setting. J Dev Behav Pediatr.

[6] World Health Organization (WHO) (2011). Guidelines on HIV disclosure counselling for children up to 12 years.

[7] National Department of Health, South Africa (2016). Disclosure Guidelines for Children and Adolescents in the context of HIV, TB and non-communicable diseases.

[8] Tomori C, Risher K, Limaye RJ, Van Lith L, Gibbs S, Smelyanskaya M, Celentano DD (2014). A role for health communication in the continuum of HIV care, treatment, and prevention. J Acquir Immune Defic Syndr.

[9] Vreeman RC, Gramelspacher AM, Gisore PO, Scanlon ML, Nyandiko WM (2013). Disclosure of HIV status to children in resource-limited settings: a systematic review. J Int AIDS Soc.

[10] Mahloko JM, Madiba SE (2012). Disclosing HIV diagnosis to children in Odi district, South Africa: reasons for disclosure and non-disclosure. Afr J Prim Health Care Fam Med.

[11] Johnson RB, Onwuegbuzie AJ (2004). Mixed methods research: a research paradigm whose time has come. Educational Researcher, 2004.

[12] Pope C, Mays N (2000). Qualitative research in health care.

[13] Rwemisisi J, Wolff B, Coutinho A, Grosskurth H, Whitworth J (2008). What if they ask how I got it? Dilemmas of disclosing parental HIV status and testing children for HIV in Uganda. Health Policy Plan.

[14] Vaz L, Eng E, Maman S, Tshikandu T, Behets F (2010). Telling children, they have HIV: lessons learned from findings of a qualitative study in sub-Saharan Africa. AIDS Patient Care STDS, 2010.

[15] Federal Republic of Nigeria (2009). Legal notice on publication of 2006 census final results. Federal Republic of Nigeria Official Gazette.

[16] National Agency for the Control of AIDS (NACA) (2015). Global AIDS Response Progress Country Report, Nigeria.

[17] Cromer B, Kliegman RM, Stanton B, Geme JS, Schor NF, Behrman RE (2015). Adolescent physical and social development. Nelson textbook of pediatrics.

[18] Braun V, Clarke V (2006). Using thematic analysis in psychology. Qual Res Psychol.

[19] Brown BJ, Oladokun RE, Osinusi K, Ochigbo S, Adewole IF, Kanki P (2011). Disclosure of HIV status to infected children in a Nigerian HIV care programme. AIDS Care.

[20] Ubesie AC, Iloh KK, Emodi IJ, Ibeziako NS, Obumneme-Anyim IN, Iloh ON (2016). HIV status disclosure rate and reasons for non-disclosure among infected children and adolescents in Enugu, Southeast Nigeria. Sahara J.

[21] Odiachi A, Abegunde D (2016). Prevalence and predictors of pediatric disclosure among HIV-infected Nigerian children on treatment. AIDS Care.

[22] Kallem S, Renner L, Ghebremichael M, Painsil E (2011). Prevalence and pattern of disclosure of HIV status in HIV infected children in Ghana. AIDS Behav.

[23] Atwiine B, Kiwanuka J, Musinguzi N (2014). Understanding the role of age in HIV disclosure rates and patterns for HIV infected children in South-Western Uganda. AIDS Care.

[24] Vreeman RC, Scanlon ML, Mwangi A (2014). A cross-sectional study of disclosure of HIV status to children and adolescents in Western Kenya. PLoS One.

[25] Mamburi LP, Hamel BC, Philemon RN (2014). Factors associated with HIV status disclosure to HIV infected children receiving care at Kilimanjaro Christian medical Centre in Moshi, Tanzania. Pan AfrMed J.

[26] Biadgilign S, Deribew A, Amberbir A (2011). Factors associated with HIV/AIDS diagnostic disclosure to HIV infected children receiving HAART: a multicentre study in Addis Ababa, Ethiopia. PLoS ONE.

[27] Moodley K, Myer L, Michaels D (2006). Paediatric HIV disclosure in South Africa- caregivers’ perspectives on discussing HIV with infected children. S Afr Med J.

[28] Britto C, Mehta K, Thomas R, Shet A (2016). Prevalence and correlates of HIV disclosure among children and adolescents in low- and middle-income countries: a systematic review. J Dev Behav Pediatr.

[29] Mellins CA, Brackis-Cott E, Dolezal C (2002). Patterns of HIV status disclosure to perinatally HIV-infected children and subsequent mental health outcomes. Clin Child Psychol Psychiatry.

[30] Wiener L, Mellins CA, Marhefka S, Battles HB (2007). Disclosure of an HIV diagnosis to children: history, current research, and future directions. J Dev Behav Pediatr.

[31] Santamaria EK, Dolezal C, Marhefka SL, Hoffman S, Ahmed Y, Elkington K (2011). Psychosocial implications of HIV sero-status disclosure to youth with perinatally acquired HIV. AIDS Patient Care STDs.

[32] John-Stewart G, Wariua G, Beima-Sofie K, Richardson B, Farquhar C, Maleche-Obimbo E (2013). Prevalence, perceptions and correlates of pediatric HIV disclosure in an HIV treatment program in Kenya. AIDS Care.

[33] Federal Ministry of Health (2010). Nigeria national guidelines for paediatric HIV and AIDS treatment and care.

[34] Tindyebwa D, Kayita J, Musoke P, Eley B, Nduati R, Tumwesigye N (2011). Kampala, Uganda: ANECCA secretariat; handbook on Paediatric AIDS in Africa by the African network for the Care of Children Affected by HIV/AIDS (ANNECA).

[35] Lesch A, Swartz L, Kagee A, Moodley K, Kafaar Z, Myer M (2007). Paediatric HIV/AIDS disclosure: towards a developmental and process-oriented approach. AIDS Care.

[36] Osingada CP, Okuga M, Nabriye RC, Sewankambo NK, Nakanjako D (2016). Prevalence, barriers and factors associated with parental disclosure of their HIV positive status to children: a cross-sectional study in an urban clinic in Kampala, Uganda. BMC Public Health.

[37] Madiba S, Mahloko J, Mokwena K (2013). Prevalence and factors associated with disclosure of HIV diagnosis to infected children receiving antiretroviral treatment in public health care facilities in Gauteng, South Africa. J Clin Res HIV AIDS Prev.

[38] Madiba S, Mokgatle M (2017). Fear of stigma, beliefs and knowledge about HIV are barriers to early access to HIV testing and disclosure for perinatally infected children and adolescents in rural communities in South Africa. S Afr Fam Pract.

[39] Kiwanuka J, Mulogo E, Haberer JE (2014). Caregiver perceptions and motivation for disclosing or concealing the diagnosis of HIV infection to children receiving HIV care in Mbarara, Uganda: a qualitative study. PLoS ONE.

[40] Fatoki B (2017). Understanding the causes and effects of stigma and discrimination in the lives of people living with HIV/AIDS: qualitative study. J AIDS Clin Res.

[41] HIV leadership through accountability programme: GNP+, NEPWHAN (2011). PLHIV stigma index Nigeria country assessment; 2011.Amsterdam: GNP+. Available from https://www.stigmaindex.org>files>reports (Accessed 26 November 2018).

[42] Bikaako-Kajura W, Luyirika E, Purcell DW, Downing J, Kaharuza F, Mermin J (2006). Disclosure of HIV status and adherence to daily drug regimens among HIV-infected children in Uganda. AIDS Behav.

[43] Corneli A, Vaz L, Dulyx J, Omba S, Rennie S, Behets F (2009). The role of disclosure in relation to assent to participate in HIV-related research among HIV-infected youth: a formative study. J Int AIDS Soc.

