# Supplementary material for: “What will my child think of me if he hears I gave him HIV?”: a sequential, explanatory, mixed-methods approach on the predictors and experience of caregivers on disclosure of HIV status to infected children in Gombe, Northeast Nigeria
Source: BMC Public Health. 2020 Mar 20;20:373. doi: 10.1186/s12889-020-08506-x (PMC7085175; doi:10.1186/s12889-020-08506-x)
Supplement: Supplementary file 1 — Additional file 1. Pretested quantitative data collection questionnaire [file 12889_2020_8506_MOESM1_ESM.pdf]

Additional file 1: pretested quantitative questionnaire

The Paediatric HIV disclosure study: a sequential, explanatory, mixed methods approach to explore predictors and caregivers' experiences on disclosure to children living with HIV in Gombe, northeast Nigeria.

Code: |__|__|__|__|

Child's Biodata

1. Age of your Child as at their last birthday (years): |__|__|
2. Sex of Child: |__|__|__|__|__|__|
3. Ethnicity: |__|__|__|__|__|__|__|__|__|__|
4. Is your child attending school? *Yes* |__| *No* |__|

Caregiver's Biodata

5. Caregiver's Religion: |__|__|__|__|__|__|__|__|__|__|__|__|
6. Caregiver's sex: |__|__|__|__|__|__|__|__|__|__|
7. Caregiver's age as at your last birthday: |__|__|
8. Caregiver's highest level of education: |__|__|__|__|__|__|__|__|__|
9. Caregiver's relationship with Child: *Biological Parent* |__| *Others (Specify)* |__|__|__|__|__|__|__|__|
10. Caregiver's HIV status: *Positive* |__| *Negative* |__| *Unknown* |__|

Caregivers opinions on HIV disclosure to children

11. Do you think children should be told their HIV status? *Yes* |__| *No* |__| Unsure |__|
12. What age (in years) do you think is appropriate to tell children about their HIV status: |__|__|
13. Do you think there are benefits of telling children their HIV status? *Yes* |__| *No* |__| Unsure |__|

Child's HIV-related clinical information

14. How old (in years) was your child when he/she was first diagnosed with HIV? |__|__|
15. Is your child on any HIV medicines? *Yes* |__| *No* |__|
16. If **Yes**, how long (in years) has your child been on HIV medicines? |__|__|
17. Has your child's HIV status been disclosed to them? (*i.e. if they been told they have HIV, or the word HIV or AIDS has been used in a discussion regarding their illness*) *Yes* |__| *No* |__|
18. If **Yes**, who told him/her about their HIV status for the first time? Mother |__| Father |__| Healthcare worker |__| Others (specify) |__|__|__|__|__|__|__|__|
19. At what age (in years) was your child told about their HIV status for the first time? |__|__|
