# Supplementary material for: “What will my child think of me if he hears I gave him HIV?”: a sequential, explanatory, mixed-methods approach on the predictors and experience of caregivers on disclosure of HIV status to infected children in Gombe, Northeast Nigeria
Source: BMC Public Health. 2020 Mar 20;20:373. doi: 10.1186/s12889-020-08506-x (PMC7085175; doi:10.1186/s12889-020-08506-x)
Supplement: Supplementary file 2 — Additional file 2. Qualitative interview guide [file 12889_2020_8506_MOESM2_ESM.pdf]

Additional file 2: Qualitative Interview Guide

RESEARCH TOPIC: *The Paediatric HIV disclosure study: a sequential, explanatory, mixed methods approach to explore predictors and caregivers' experiences on disclosure to children living with HIV in Gombe, northeast Nigeria.*

CODE NO: |__|__|__|__|

Sociodemographic characteristics of caregiver:

Caregivers' sex: ______ Age: |__|__| HIV Status: _____ Relationship to child: _______

Caregivers' Education: _____Child's sex: ________ Child's age: |__|__|

Interview questions

1. Could you tell me about your own experience about the process of making disclosure to HIV positive children?
2. What is your opinion concerning informing / telling a HIV positive child about his/her HIV positive status?
3. Are there reasons why children with HIV should not be informed about their status?
4. From your experience, whose responsibility is it to inform an HIV positive infected child bout his / her status?
